# Adolescent Fat Embolism Syndrome after Closed Tibial Shaft Fracture: Treatment with Emergent External Fixation

**DOI:** 10.1155/2021/5585085

**Published:** 2021-04-24

**Authors:** Dillon C. O'Neill, Graham J. Dekeyser, Alexander J. Mortensen, Christopher A. Makarewich

**Affiliations:** ^1^Department of Orthopaedic Surgery, University of Utah, Salt Lake City, UT, USA; ^2^School of Medicine, University of Utah, Salt Lake City, UT, USA; ^3^Department of Orthopaedic Surgery, University of Utah, Salt Lake City, UT, USA

## Abstract

**Case:**

An adolescent male developed fat embolism syndrome 24 hours after sustaining a closed right tibial shaft fracture in a football game. The patient was treated with emergent external fixator application due to declining respiratory and mental status and experienced swift recovery after stabilization. He was treated with an intramedullary nail within 1 week of injury.

**Conclusion:**

Pediatric fat embolism syndrome is uncommon, and a high index of suspicion is required to facilitate appropriate orthopaedic involvement. External fixation can be performed emergently with minimal fracture manipulation. Rapid provisional fixation appears to have facilitated recovery in this example.

## 1. Introduction

Fat embolism syndrome (FES) results from an inflammatory response to embolized fat and intramedullary contents in the systemic and pulmonary circulation, and most commonly occurs 24-72 hours following trauma to lower extremity long bones [1, 2]. Characterized by neurocognitive dysfunction, respiratory distress, and right heart strain, FES has been estimated to be fatal in 5-20% of patients [2]. There is limited literature on FES in pediatric and adolescent patients.

We present the case of a 17-year-old patient who developed FES and associated acute hypoxic respiratory failure in the setting of an isolated closed tibial shaft fracture. The patient was emergently stabilized with external fixation, followed by rapid improvement in both respiratory and neurologic function, an outcome which, to our knowledge, has not previously been documented in the adolescent population.

At the time of manuscript preparation, the patient was 17 years old. He was informed that data concerning the case would be submitted for publication, and both he and his parents provided consent.

## 2. Case Report

**Figure 1:**
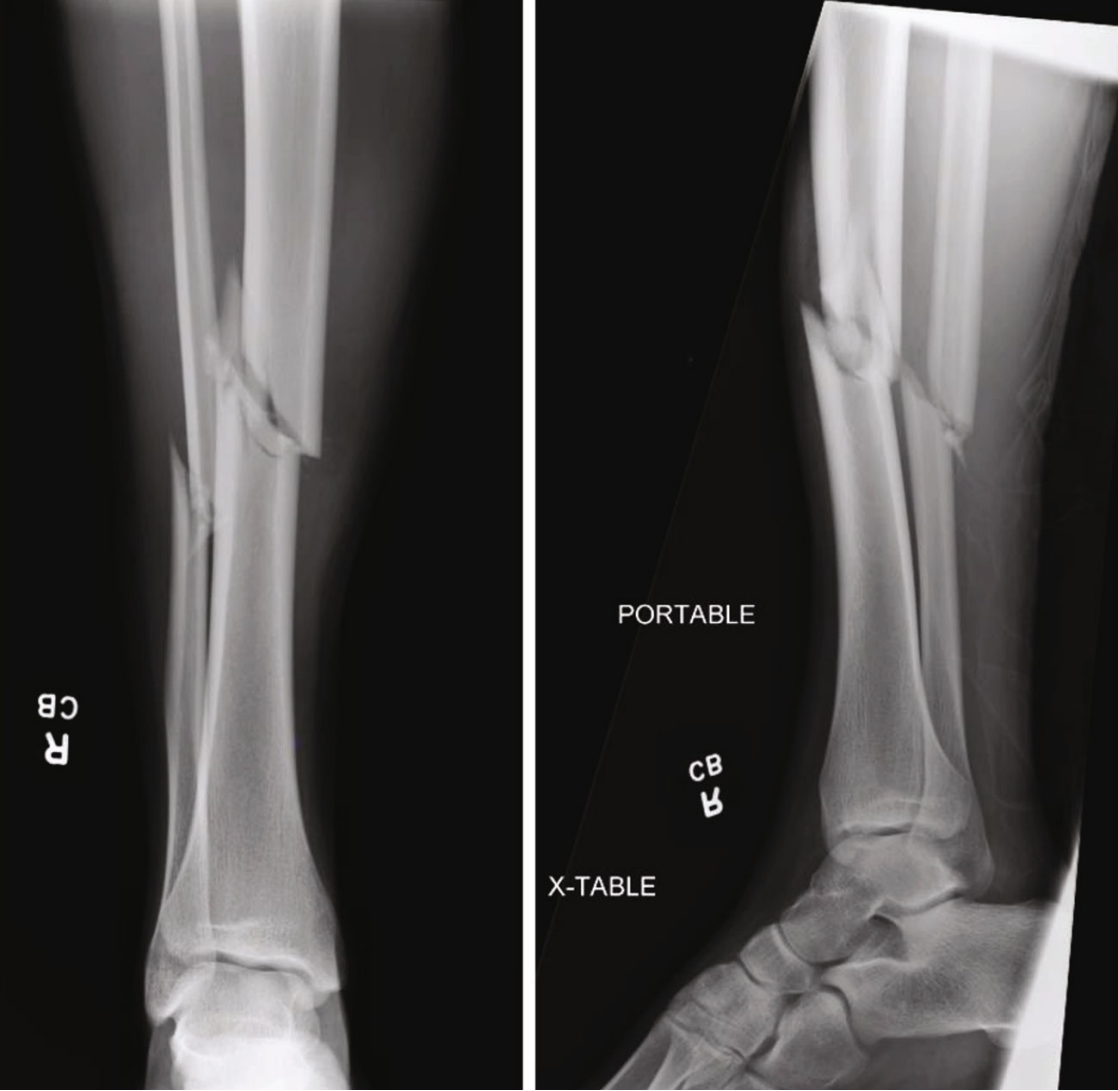
Injury radiographs. AP and lateral radiographs of the right tibia and fibula obtained at an outside hospital emergency department demonstrated a short oblique diaphyseal tibia fracture with mild comminution.

A 17-year-old male sustained right midshaft tibia and fibula fractures in a high school football game. The patient had previously been healthy without significant past medical history. The patient was transported to a local hospital where radiographs demonstrated a short oblique fracture of the tibial diaphysis with mild comminution (AO/OTA classification 42-B2) [3] (Figure 1). At the outside emergency department, the patient was placed in a long leg splint and discharged home with planned definitive fixation the following morning.

**Figure 2:**
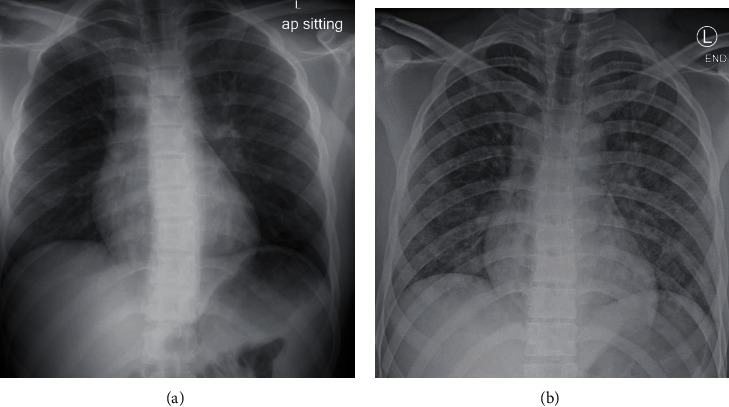
Chest radiographs. (a) Chest radiograph from the morning after right tibia fracture demonstrates mild, diffuse ground-glass opacities. (b) Chest radiograph from the time of rapid response for worsening neurocognitive and respiratory status demonstrates worsening diffuse pulmonary infiltrates.

Following discharge, the patient experienced a syncopal episode at home and returned to his local emergency department. On arrival, he was found to be hypoxic, requiring 1-3 L/min of low flow nasal cannula (LFNC) oxygen to maintain saturation. A chest radiograph demonstrated mild bilateral interstitial and alveolar opacities (Figure 2). CT of the chest was notable for scattered ground-glass opacities. The patient tested negative for COVID-19. Given the patient's fracture history, respiratory symptoms, and chest imaging, the pulmonology department at this hospital recommended the patient be transferred for a higher level of care for suspected FES.

The patient was admitted to the pediatrics service at our level I pediatric trauma center at approximately 2000, 24 hours postinjury. He was febrile to 38.0°C and requiring 3 L/min LFNC to maintain saturations. Otherwise, vital signs were within normal limits. The patient was at his baseline mental status. The orthopaedic surgery service was first notified about the patient once he had arrived in the hospital, and the orthopaedic resident on-call was present at the bedside at approximately 2100. On exam, the patient was now requiring 10 L/min via simple mask to maintain oxygen saturation in the mid-90s. Examination of the right lower extremity demonstrated diffuse swelling about the lower leg without concern for open fracture or compartment syndrome. The patient's splint wrapping was taken down and re-wrapped during the examination, but the splint was not removed.

A rapid response was called one hour after orthopaedic examination due to deteriorating respiratory status and new mental status changes. The patient was transferred to the pediatric intensive care unit (PICU) in acute hypoxic respiratory failure requiring continuous positive airway pressure (CPAP) at 50% FiO_2_ to maintain saturations. Repeat chest radiograph demonstrated worsening interstitial opacities (Figure 2). The orthopaedic service was not notified about the patient's transfer to the PICU and found out the patient had been transferred when the resident on-call presented to the patient's original hospital room in order to reassess the patient.

After discussion with the PICU team, who forecasted that the patient was likely to be intubated regardless of whether or not emergent operative intervention was pursued, the decision was made to provisionally stabilize the patient's fracture in an attempt to prevent further fat embolization. The patient was taken to the operating room emergently approximately 6 hours after arrival to our hospital and 3 hours after mental status changes and respiratory distress.

**Figure 3:**
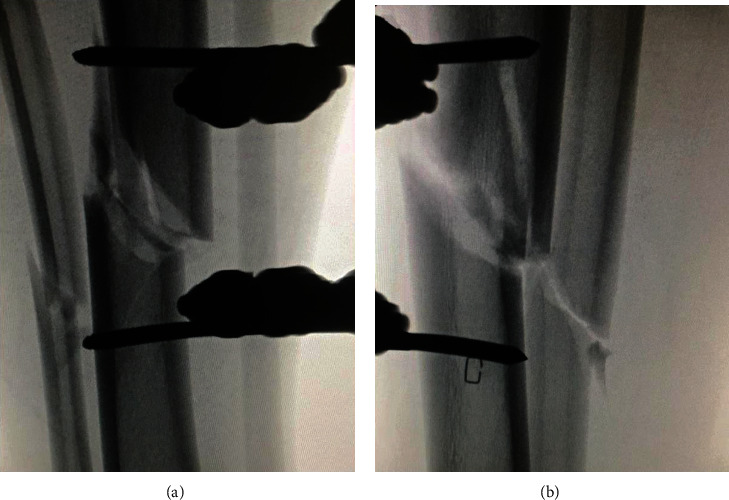
Fluoroscopic fracture alignment following uniplanar external fixator application: (a) anteroposterior view; (b) lateral view.

**Figure 4:**
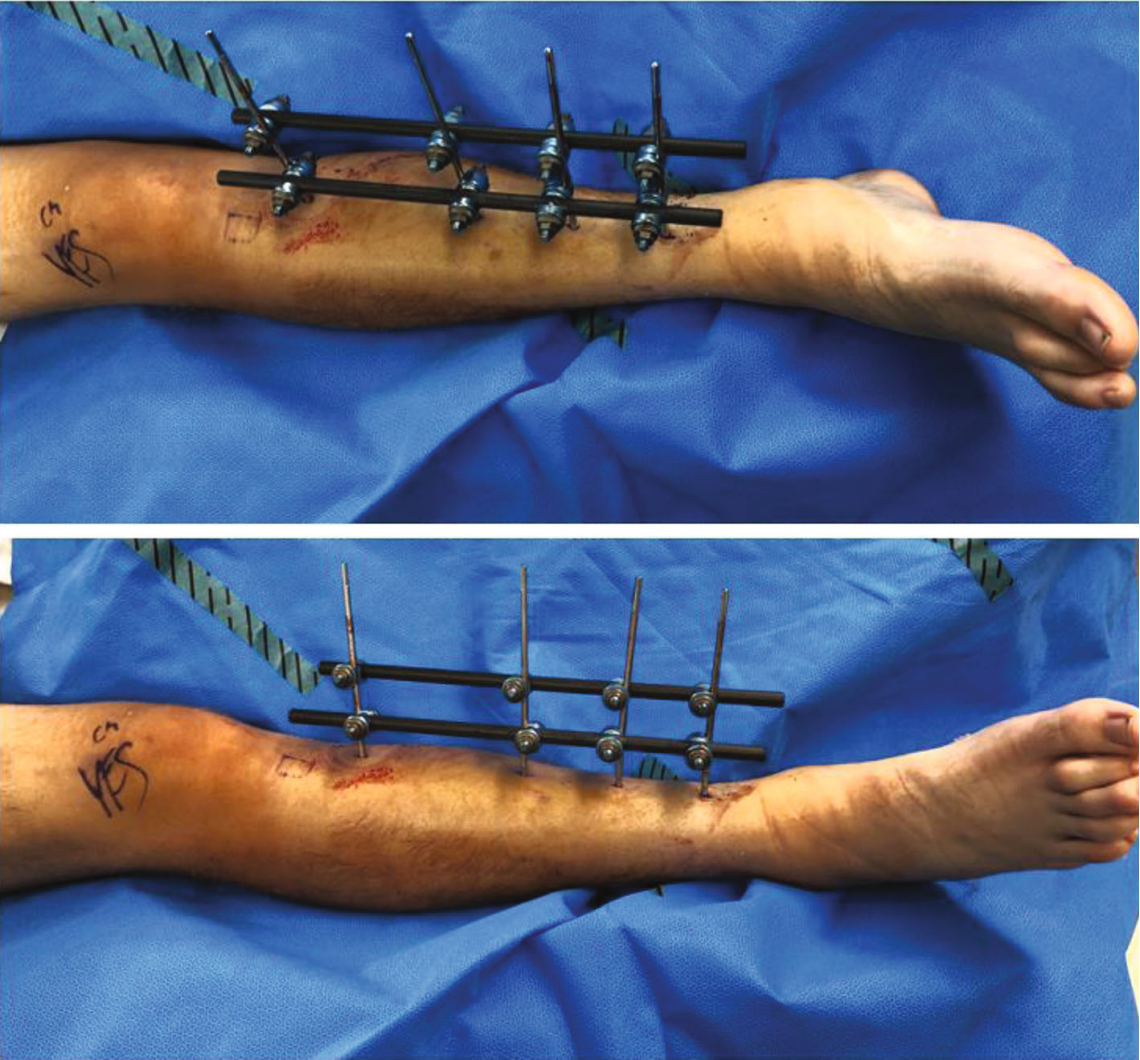
Clinical photographs of patient's external fixator construct taken at the time of definitive fixation on postoperative day 4.

After the patient had been intubated on his hospital bed, he was carefully transferred to the operating table with his splint in place. With attention to minimize movement at the fracture site, the right lower extremity splint wrappings were removed with scissors. The anterior tibia was prepped with chlorhexidine and draped while keeping the splint in place and without manipulating the leg. A uniplanar external fixator was then applied with fluoroscopic guidance (Figures 3 and 4).

**Figure 5:**
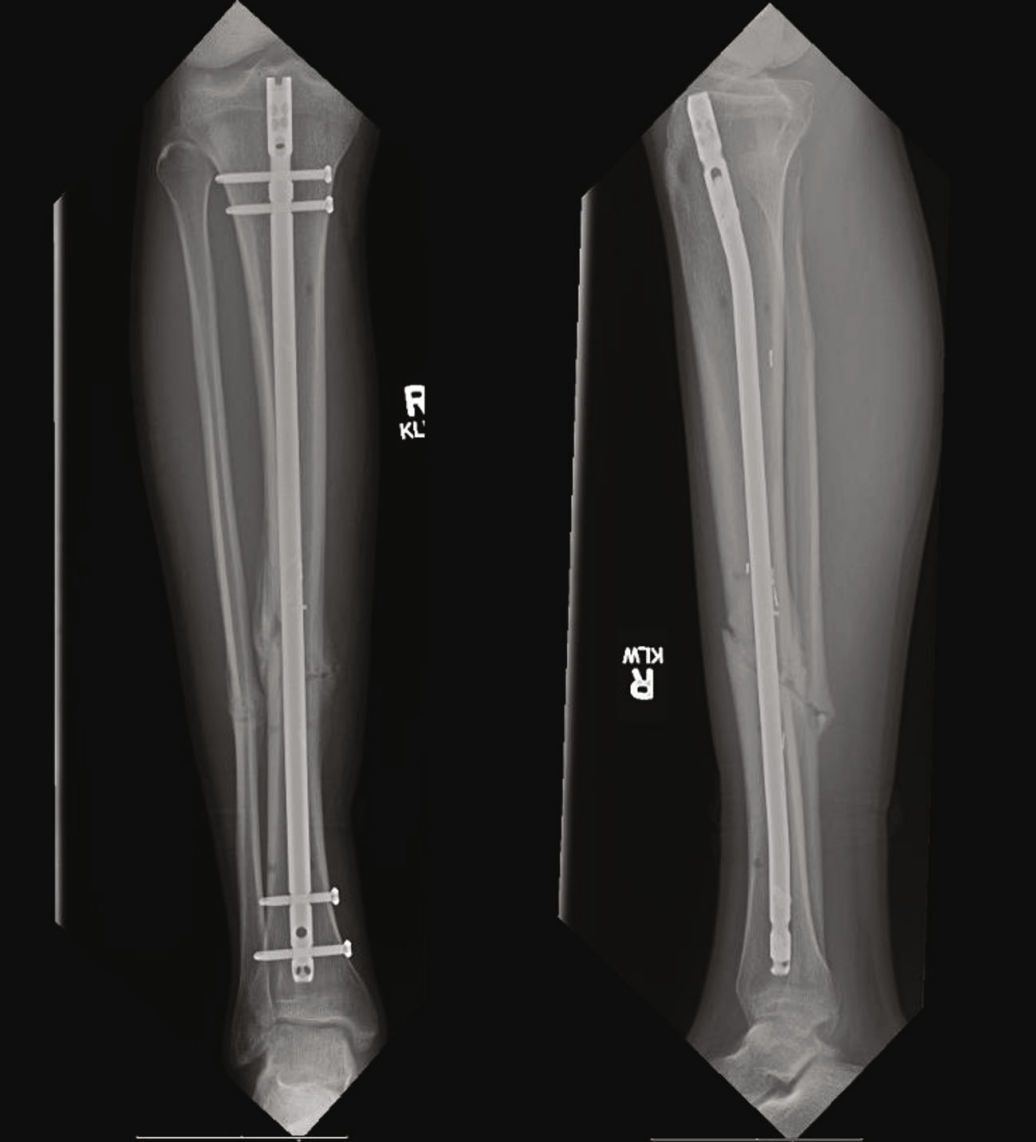
Three-month postoperative radiographs. AP and lateral radiographs at three months postoperatively demonstrate fracture union.

Following surgery, the patient was transferred to the PICU intubated. He was extubated approximately 12 hours postoperatively. His mental status had returned to baseline at the time of extubation. He was weaned from supplemental oxygen by the evening of postoperative day 3. He underwent intramedullary nailing using a reamer/irrigator/aspirator graft harvesting system (RIA 2, Synthes, West Chester, PA) to minimize fat embolization during definitive fixation on the afternoon of postoperative day 4. He was discharged from the hospital in good health on postoperative day 5. Three months postoperatively, the patient had returned to full activity and was without complaint. Three-month postoperative radiographs demonstrated fracture union (Figure 5).

## 3. Discussion

The classic presentation of FES includes the triad of respiratory distress, neurological symptoms, and a characteristic petechial rash [4], which are thought to be caused by combination of two incompletely understood mechanisms: mechanical obstruction and biochemical injury [5, 6]. The mechanical obstruction theory describes the uptake of intramedullary fat into venous circulation, which attracts platelets and accelerates fibrin formation, and eventually leads to pulmonary capillary obstruction with resultant hypoxemia. The biochemical theory describes a proinflammatory state in response to products of fat metabolism, including free fatty acids and glycerol, which trigger the release of a damaging cytokine cascade [5, 6].

An emphasis has been placed on the prevention of FES for multiple reasons [2]. Recognition and prompt diagnosis of FES is clinically challenging, and without effective targeted treatment options, the current management of FES primarily consists of supportive care. Several studies support early fracture stabilization as a means of reducing the risk of developing FES [7–10]. However, the literature is lacking high-level evidence to support specific timing of definitive fixation for major long bone fractures with regard to the prevention of FES [11]. The severity of FES is thought to be directly related to the total amount of embolized fat and indirectly related to the cardiopulmonary reserve of a patient [5, 12]. Therefore, early fracture reduction and stabilization may theoretically play a role in decreasing the total amount of intramedullary fat embolization—effectively reducing the severity of FES.

In addition to timing of fracture fixation, the optimal construct used for fracture stabilization in the prevention of FES is unknown. Research has shown canal pressurization through intramedullary reaming and nailing increases the showering of fat emboli into venous circulation [12, 13], making it reasonable to avoid these techniques early in the course of FES in an attempt to minimize the risk of exacerbating cardiopulmonary distress [11, 14]. However, clinical research has not proven that intramedullary techniques place patients at increased risk of respiratory distress [15]. External fixation of long bone fractures has been shown to result in reduced intramedullary pressurization and less fat embolization compared to long bone reaming and nailing while providing acceptable treatment outcomes [16, 17]. The theory of damage control orthopaedics suggests the rapid and rigid stabilization provided by external fixation is an effective bridge to definitive fixation while minimizing complications in multiply injured patients [18, 19]. In the context of FES secondary to traumatic lower extremity long bone fracture, external fixation may play a similar “damage control” role in temporarily providing rigid fracture stability without pressurization of the intramedullary canal in order to minimize fat emboli showering and reduce the severity of FES [20, 21].

The current clinical example provides anecdotal support of the above research. In this case, emergent provisional fixation using an external fixator provided sufficient stability to minimize further embolic insult and the patient improved rapidly following provisional fixation. In addition, this case demonstrates the feasibility of sterile ex-fix application with minimal fracture manipulation at the time of provisional stabilization.

Patients aged 10-39 years old are at the highest risk of developing posttraumatic FES, with a relative risk of 7.5 times greater than patients aged 40 and older [1, 22, 23]. Pediatric patients aged 10-18 years old fall within this high-risk age range while children < 10 years old have low rates of FES. This difference has been attributed to the transition from primarily hematopoietic bone marrow early in life to increasingly fatty bone marrow with advancing age [1, 24]. Consequently, substantially less is known about FES in young children, with case reports and small case series providing the majority of literature [25, 26]. While adolescent fat embolism syndrome is less rare, literature often combines adolescents with older adults rather than studying adolescent groups in isolation [1, 22, 23, 27]. As such, no clear guidelines exist for the management of fat embolism syndrome in pediatric patients. This was apparent in the current clinical example. If not for advocacy from orthopaedics, emergent operative fixation for this patient would not have been considered by the primary services involved, highlighting the importance of close orthopaedic involvement in any case of suspected pediatric fat embolism syndrome.

## Data Availability

There is no data to be made available.
